# Trajectory of early postoperative nutritional status and its predictors in patients with gastrointestinal cancer: a longitudinal study

**DOI:** 10.3389/fnut.2026.1790657

**Published:** 2026-06-23

**Authors:** Jie Chen, Chunhua Zhang, Liuliu Zhang, Hui Wang, Gaoyue Dong, Zeng Chen, Youxiang Bu, Yanfei Jin, Liu Yang, Zhiyan Zhou, Jun Yao, Xiaoxu Zhi

**Affiliations:** 1 The Affifiliated Cancer Hospital of Nanjing Medical University and Jiangsu Cancer Hospital and Jiangsu Institute of Cancer Research, Nanjing, China; 2 School of Nursing, Nanjing Medical University, Nanjing, China; 3 School of Health Policy and Management, Nanjing Medical University, Nanjing, China; 4 Institute of Healthy Jiangsu Development, Nanjing Medical University, Nanjing, China

**Keywords:** gastrointestinal cancer, latent class growth model, longitudinal studies, nutritional status, surgery

## Abstract

**Objective:**

To explore the trajectory categories of early postoperative nutritional status in patients with gastrointestinal cancer and to analyze their predictive factors using both multiple logistic regression and decision tree models, providing a basis for implementing precise nutritional interventions.

**Methods:**

A longitudinal study design was used, and 351 patients who underwent radical surgery for gastrointestinal cancer were conveniently selected as the study subjects. Nutritional status was assessed using the Patient-Generated Subjective Global Assessment (PG-SGA) at four time points: preoperatively (T1), 1 week postoperatively (T2), 1 month postoperatively (T3), and 3 months postoperatively (T4). Latent class growth modeling (LCGM) was employed to identify different nutritional status trajectory categories. Multiple logistic regression and CHAID decision tree models were used to analyze the predictive effects of demographic characteristics, disease characteristics, sense of coherence, and social support on trajectory categories.

**Results:**

Three distinct nutritional status trajectory categories were successfully identified: the “Moderate Malnutrition-Gradual Recovery” group (40.5%), the “Persistent Severe Malnutrition” group (11.7%), and the “Transient Moderate Malnutrition-Rapid Recovery” group (47.9%). Both multiple logistic regression and decision tree models confirmed that cancer type (esophageal cancer), low handgrip strength, and low level of sense of coherence were core predictive factors affecting trajectory categories. Additionally, logistic regression identified male and low level of social support as independent risk factors, while the decision tree model further revealed key pathways for identifying specific high-risk subgroups based on factors such as chemotherapy history, neoadjuvant therapy, age, and surgical approach.

**Conclusion:**

Early postoperative nutritional status trajectories in patients with gastrointestinal cancer show heterogeneity. Integrating the two predictive models can create a more comprehensive risk assessment framework, allowing preliminary screening using factors identified by logistic regression and targeted preventative management for key high-risk groups based on pathways highlighted by the decision tree model.

## 1 Introduction

Gastrointestinal cancers, such as esophageal cancer, gastric cancer, and colorectal cancer, pose a major global public health challenge, with a heavy burden of both incidence and mortality. According to the latest data from the International Agency for Research on Cancer (IARC), in 2022, the number of new colorectal cancer cases worldwide (1.92 million) ranked third globally, while gastric cancer (960,000) and esophageal cancer (510,000) ranked fifth and eleventh, respectively. In terms of mortality, these cancers ranked second, fifth, and seventh among all cancer types (1). In China, colorectal cancer and gastric cancer ranked second in terms of new cases and third in mortality, respectively (2); new cases and deaths from esophageal cancer accounted for 43.8 and 42.1% of the global totals, significantly higher than the global average (3). Due to the unique location of these tumors, patients commonly face eating difficulties, impaired digestive and absorptive function, and treatment-related toxicities, resulting in a high incidence of malnutrition. Numerous studies have shown that the nutritional status of patients with gastrointestinal cancer is concerning. A survey by Song et al. (4) indicated that the prevalence of malnutrition in gastrointestinal cancer patients ranged from 80 to 93.7%, with gastric cancer patients being the most affected (92.7%), among whom 41.3% suffered from severe malnutrition. Research by Yang et al. (5) further confirmed that the overall prevalence of malnutrition in gastrointestinal cancer patients was 80.6%, with severe malnutrition accounting for 34.2%. In addition to the high prevalence of malnutrition, its profound impact on clinical outcomes further underscores the urgency of nutritional management. Malnutrition not only delays surgical recovery and prolongs hospital length of stay, but also significantly increases hospital costs, posing a substantial burden on healthcare systems and health policy planning (6).

At present, most studies have focused on the cross-sectional prevalence of malnutrition, while dynamic nutritional trajectory analysis—which captures individual variability over time—remains largely underexplored. This gap highlights the imperative for trajectory-based approaches. In particular, the early postoperative period presents additional challenges as a critical vulnerable phase and a ‘low point’ for the nutritional status of patients with gastrointestinal cancers. Due to multiple factors such as surgical trauma stress, inability to eat normally, changes in digestive tract anatomy, and metabolic disorders, patients’ nutritional status deteriorates during this stage. Studies have shown that 1 week and 1 month after surgery are peak periods for both the incidence and severity of malnutrition (7, 8). Nevertheless, there is substantial heterogeneity in nutritional status trajectories. Guo et al. (9) found that there are three types of BMI change trajectories in elderly postoperative gastric cancer patients: slow decline, rapid decline, and stable BMI. The heterogeneity observed in these trajectories suggests that focusing only on overall trends while ignoring individual differences may lead to a misallocation of intervention resources. Accurately characterizing the early postoperative nutritional trajectories of patients with gastrointestinal cancers is of great significance for identifying high-risk subgroups and achieving precise nutritional support.

Against this backdrop, exploring modifiable influencing factors has become key to optimizing nutritional management. Previous studies have largely focused on demographic and disease-related factors, with relatively insufficient attention given to psychosocial factors. In fact, psychosocial factors profoundly affect patients’ willingness to eat, nutritional behaviors (9–13), and confidence in recovery, and are highly modifiable, offering a new perspective for developing multidimensional, individualized intervention strategies. This study aims to use multivariate logistic regression and decision tree machine learning methods to identify factors influencing early postoperative nutritional status trajectories in patients with gastrointestinal cancer. Decision tree models can efficiently handle complex interactions between variables and generate intuitive, easily interpretable clinical decision rules, thereby helping clinicians quickly identify high-risk populations that require prioritized intervention (14).

In summary, this study aims to characterize the dynamic trajectory of early postoperative nutritional status through longitudinal follow-up, identify subgroups with different evolution patterns, and, based on demographic and disease characteristics while incorporating factors such as sense of coherence and social support, construct predictive models using multivariate logistic regression and decision tree algorithms. By providing a scientifically grounded and clinically actionable tool, this study will support clinical decision-making, enable early identification of patients at high risk of nutritional deterioration, and facilitate the implementation of individualized nutritional interventions, ultimately improving surgical recovery, reducing hospital stays and costs, and informing health policy optimization.

## 2 Materials and methods

### 2.1 Patients

#### 2.1.1 Sampling methods and sample size

Convenience sampling was used to select patients with gastrointestinal cancer who visited a tertiary grade-A cancer hospital in Jiangsu Province from June 2022 to January 2024 as the study subjects. The key statistical method of this study is the latent growth model. There is currently limited research on the sample size required for latent class analysis (LCA), and no universally accepted formula exists for its calculation. Therefore, the study referred to the empirical standards commonly adopted in similar research, which generally recommend a sample size greater than 200 (15). In addition, some studies have borrowed sample size estimation methods from cross-sectional designs, suggesting that the sample size should be 5 to 10 times the number of variables. Yang approached the issue from the perspective of model selection accuracy and proposed that each latent class should include at least 50 participants to ensure correct model identification based on the aBIC. Given that the number of latent classes typically does not exceed 5, the minimum required sample size is approximately 250 (16). Considering all of the above criteria, the study ultimately enrolled 370 patients for follow-up, with an effective analytical sample of 351 cases, which satisfied all the aforementioned requirements. A longitudinal study design was adopted to assess the nutritional status of patients with gastrointestinal cancer preoperatively (T1), 1 week postoperatively (T2), 1 month postoperatively (T3), and 3 months postoperatively (T4). Taking into account the distribution of cancer types and potential sample attrition, 370 patients were selected for follow-up preoperatively in this study. The study was conducted in accordance with the Declaration of Helsinki, and written informed consent was obtained from each participant. The study was approved by the hospital ethics committee (Ethics No.: 2022Ke-Kuai058).

#### 2.1.2 Inclusion and exclusion criteria

Inclusion criteria: ① Pathologically diagnosed with gastrointestinal cancer, aged 18–80 years; ② Undergoing radical cancer surgery for the first time; ③ Expected survival of more than 1 year; ④ Provided informed consent to participate in this study. Exclusion criteria: ① Mental or cognitive disorders preventing communication; ② Severe organic diseases (cardiovascular or cerebrovascular diseases, liver or kidney failure, or concurrent other tumors, etc.). Elimination criteria: ① Occurrence of severe complications or disease progression during follow-up; ② Withdrawal from the study for personal reasons (refusal to participate, loss to follow-up); ③ Completion of fewer than three surveys.

### 2.2 Research tools

#### 2.2.1 General information questionnaire

The general information questionnaire is designed based on a review of the literature and includes demographic information (age, gender, educational level, employment status, etc.) and disease-related information (serum albumin, handgrip strength, comorbidities, surgical approach, etc.). Specifically, serum albumin was measured from fasting venous blood using the bromocresol green method (reference range 40–55 g/L). Handgrip strength was measured using an electronic dynamometer (three trials, maximum recorded), with the patient seated, elbow at 90°, in accordance with the American Society of Hand Therapists standard (17). Comorbidities were defined as the presence of any chronic condition (e.g., hypertension, diabetes, coronary heart disease, COPD, sequelae of stroke), recorded as ‘yes’ or ‘no’. The surgical approach was categorised as endoscopic or open surgery based on surgical records.

#### 2.2.2 Patient-generated subjective global assessment, PG-SGA

The PG-SGA was adapted by Ottery (18) based on the Subjective Global Assessment scale and is used to evaluate the nutritional status of patients. This scale consists of two parts: patient self-assessment and healthcare professional evaluation. The patient self-assessment covers four main aspects: weight loss, dietary intake, symptoms, and activity level. The healthcare professional evaluation includes: disease status, metabolic stress, and physical examination (assessing fat, muscle, and fluid depletion). The total score is the sum of the scores from patient self-assessment, disease status, metabolic stress, and physical examination. A score of 0–1 indicates good nutrition, 2–3 suggests potential malnutrition, 4–8 indicates moderate malnutrition, and ≥9 indicates severe malnutrition. Studies have shown that using this scale for nutritional screening in cancer patients demonstrates high specificity (91%) and sensitivity (94%) (19).

#### 2.2.3 Sense of coherence-13, SOC-13

SOC-13 is a simplified version of SOC-29 developed by Antonovsky (20) and adapted into Chinese by Shiu (21). This scale contains 13 items across three dimensions: comprehensibility (corresponding to items 1–5), manageability (corresponding to items 6–9), and meaningfulness (corresponding to items 10–13). The scale uses a 7-point Likert scoring method, with items 1, 2, 3, 8, and 13 requiring reverse scoring. The total score is obtained by summing the scores of the items in each dimension, ranging from 13 to 91, where 13–63 indicates a low level, 64–79 a medium level, and 80–91 a high level. The internal consistency coefficient of the scale is 0.76, the test–retest reliability is 0.61 (22, 23), and the item-dimension structure is reasonable (24).

#### 2.2.4 Social supporting rating scale, SSRS

The SSRS was developed by Xiao (25) and others to assess the extent of social support an individual receives. The scale consists of 10 items and includes three dimensions: subjective support (corresponding to items 2, 6, and 7), objective support (corresponding to items 1, 3, 4, and 5), and the utilization of social support (corresponding to items 8, 9, and 10). Items 1–4 and 8–10 are single-choice questions, scored 1–4 points, respectively. Item 5 assesses the level of support from different family members, scored from 1 to 4 points ranging from “none” to “full support,” with the total summed. Items 6–7 are scored based on the number of support sources, with a maximum score of 9 points; if there are no support sources, 0 points are assigned. The total social support score is the sum of the item scores, with higher scores indicating higher levels of social support. Scores of 22 or below indicate low support, 23–44 indicate moderate support, and 45–66 indicate high support. The Cronbach’s *α* coefficient for the items on the scale ranges from 0.890 to 0.940.

### 2.3 Data collection methods

The investigators were clinical nurses with more than 5 years of clinical experience, who strictly selected study subjects according to the inclusion and exclusion criteria to ensure sample homogeneity. At T1, after the investigators had received unified training and passed assessment, they collected data from patients face-to-face, obtaining demographic and disease information through reviewing medical records. Prior to the survey, the investigators must explain the purpose and significance of the study to the participants and obtain their consent before distributing the questionnaires (PG-SGA, SOC-13, SSRS scales). During the survey, a standardized script was used to explain the requirements for completing the questionnaires to ensure the procedure was standardized and accurate. For participants with lower education level or writing difficulties, the investigators may assist by objectively recording responses on their behalf after thorough communication. Upon completion of the survey, the investigators collected and verified the questionnaires on site, informing the patients to supplement any missing information promptly. After the survey, patients were informed about the three follow-up time points and relevant precautions, their contact information was verified, and a WeChat group was established for patient management. At T2, T3, and T4, a combination of in-person scheduled surveys and telephone follow-ups was used to assess the patients’ nutritional status.

### 2.4 Statistical methods

Data analysis and processing were conducted using IBM SPSS 25.0 and R 4.3.1. Quantitative data conforming to a normal distribution were described using mean ± standard deviation, while categorical data were described using frequency and percentage. In this study, a latent class growth model was employed to identify subgroups of gastrointestinal cancer patients with different nutritional status trajectories, using patients’ T1-T4 PG-SGA scores as the observed variables for model fitting. Models ranging from 1 to 5 classes were sequentially fitted to determine the optimal number of classes that best represented the data characteristics. Model selection and determination were primarily based on the following statistical fit indices: ① Information criteria, including Akaike information criterion (AIC), Bayesian Information Criterion (BIC), corrected AIC (cAIC), sample size-adjusted BIC (ssaBIC), and Hannan-Quinn Information Criterion (HQIC). Lower values for these indices indicate better model fit, with BIC regarded as one of the most reliable indicators due to its stringent penalty for model complexity (26). ② Classification accuracy was assessed using the average posterior probability, with values greater than 0.7 indicating good reliability in individual classification. In this study, 64 patients were lost to follow-up due to changes in treatment regimen, severe complications, refusal to participate, or transfer to other hospitals, and ultimately, 351 patients who completed at least three assessments were included. Among these 351 patients, some had missing data at certain time points; therefore, median imputation was used to handle missing values during model fitting. After determining the trajectory classes, univariate analysis was performed using Chi-square tests, non-parametric tests, and independent two-sample *t*-tests. Variables showing statistically significant differences in the univariate analysis were subsequently included in logistic regression and decision tree models. Using the postoperative early nutritional status trajectory category as the dependent variable, and the variables showing statistically significant differences in univariate analysis as the independent variables, a CHAID decision tree model was constructed using SPSS Modeler 18.0. This algorithm employed the chi-square test of independence as the core splitting criterion, selecting the optimal split by testing the statistical significance of the association between each candidate predictor variable and the three trajectory categories; the significance level (*α*) in the model was set at 0.05. Preliminary pruning was performed by adjusting the maximum tree depth and minimum node sample size to control model complexity and prevent overfitting. Given the small sample size for Trajectory 2, and to ensure the stability of model training and the reliability of evaluation, this study did not establish a separate validation set; instead, the Hold-out Method was adopted to validate the model construction. The total sample was randomly divided into a training set (70%) and a test set (30%) for model construction and performance evaluation. In view of the multi-class outcome, the class-imbalanced data structure, and the emphasis on clinical interpretability in this study, we adopted precision, recall (sensitivity) and specificity as core evaluation metrics. These metrics provided an intuitive and clinically relevant performance assessment for each prediction category across three dimensions: predictive reliability, case detection capability, and negative exclusion capability. The final model was selected not only based on its superior discriminatory performance on the test set but also on the requirement that its decision-making pathways possess reasonable interpretability in clinical practice, thereby ensuring the model combines statistical reliability with practical application value.

## 3 Results

### 3.1 General information of patients with gastrointestinal cancers

**Figure 1:**
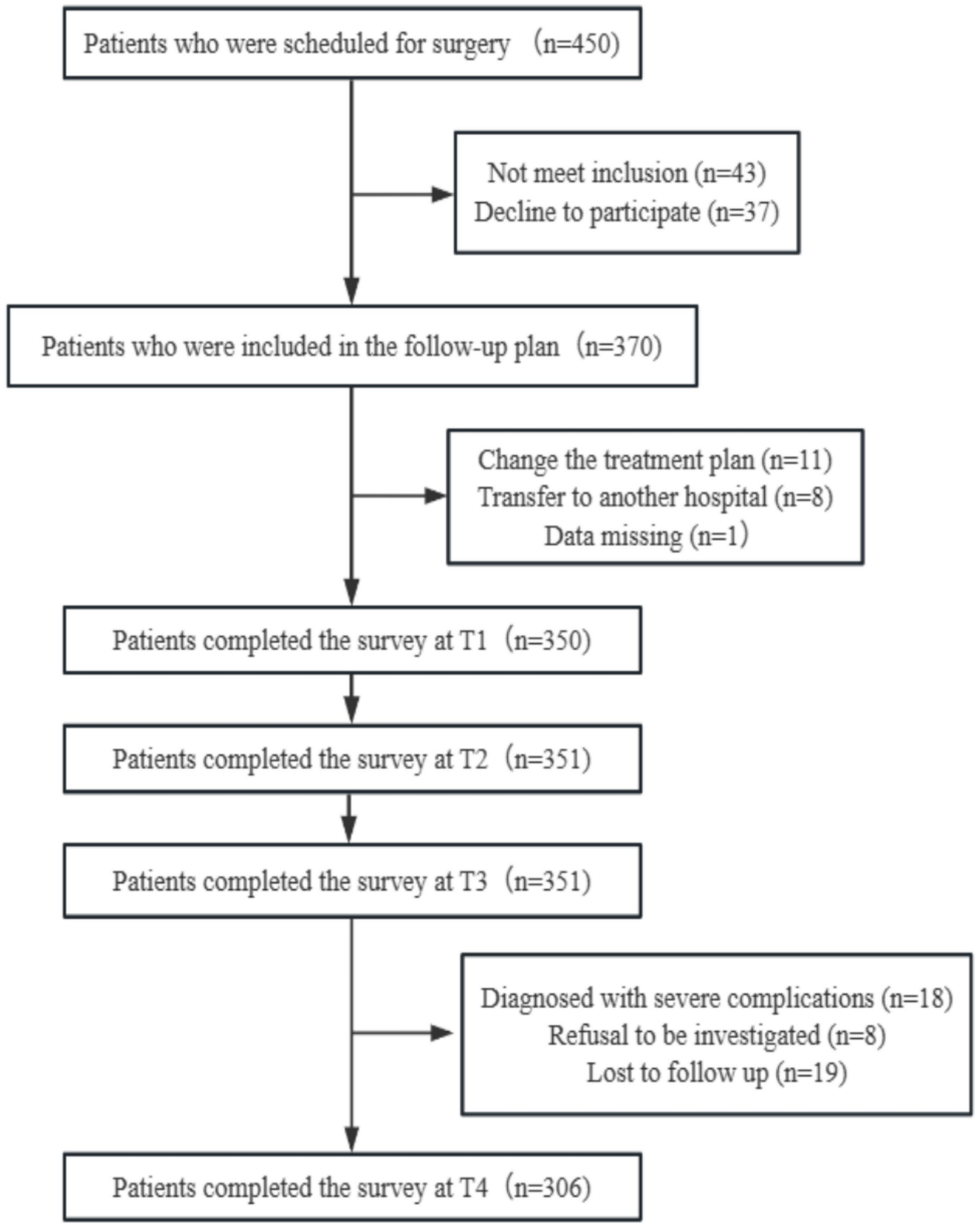
The recruitment flowchart.

We obtained a list of 450 patients awaiting surgery from the hospital system. Based on the inclusion criteria, the initial 370 patients were enrolled in the follow-up plan. Due to changes in treatment plans, refusal to participate in the survey, inability to contact after transfer, or severe complications, 64 patients were lost to follow-up, resulting in a loss rate of 17.29%. The recruitment process was detailed in Figure 1. Ultimately, a total of 351 patients who completed at least three surveys were included, with their basic information shown in Table 1.

**Table 1:** Basic information of patients in the early postoperative period of gastrointestinal cancer (*n* = 351).

Variable	Group	Case number	Percentage (%)
Gender			
	Male	253	72.08
	Female	98	27.92
Age			
	18–39	12	3.42
	40–59	118	33.62
	60–79	221	62.96
Place of residence			
	City	155	44.16
	Countryside	196	55.84
Employment status			
	Employed	105	29.91
	Retired	228	64.96
	Unemployed	18	5.13
Education level			
	Primary school and below	137	39.03
	Junior/High school or technical secondary school	177	50.43
	College and above	37	10.54
Monthly household income			
	<3,000	80	22.79
	3,000–5,000	119	33.90
	5,000–10,000	84	23.93
	>10,000	68	19.37
Medical insurance type			
	Urban and rural residents medical insurance	48	13.68
	Employee medical insurance	131	37.32
	New rural cooperative medical care	162	46.15
	Full self-payment	10	2.85
Comorbidities			
	No	155	44.16
	Yes	196	55.84
Cancer type			
	Esophageal cancer	114	32.48
	Gastric cancer	49	13.96
	Colorectal cancer	188	53.56
Cancer stage			
	I	74	21.08
	II	129	36.75
	III	109	31.05
	IV	39	11.11
Preoperative neoadjuvant therapy			
	No	310	88.32
	Yes	41	11.68
Surgical approach			
	Endoscope surgery	213	60.68
	Open surgery	138	39.32
Radiotherapy history			
	No	326	92.88
	Yes	25	7.12
Chemotherapy history			
	No	181	51.57
	Yes	170	48.43

### 3.2 Postoperative early nutritional status scores of patients with gastrointestinal cancer

The nutritional status of patients with gastrointestinal cancer at four time points, T1 to T4, was scored as (3.58 ± 2.29), (8.71 ± 3.73), (6.86 ± 3.98), and (5.61 ± 3.62), respectively, showing an overall trend of initial increase followed by a decrease. The incidence of severe malnutrition at T1 to T4 was 40.57, 97.72, 82.05, and 48.37%, respectively, as detailed in Table 2.

**Table 2:** Nutritional status scores of postoperative early-stage gastrointestinal cancer patients from T1 to T4.

Timing	PG-SGA classification *n* (%)				PG-SGA
	0–1	2–3	4–8	≥9	
T1 (*n* = 350)	46 (13.14)	162 (46.29)	132 (37.71)	10 (2.86)	3.58 ± 2.29
T2 (*n* = 351)	0 (0)	8 (2.28)	195 (55.56)	148 (42.16)	8.71 ± 3.73
T3 (*n* = 351)	4 (1.14)	59 (16.81)	190 (54.13)	98 (27.92)	6.86 ± 3.98
T4 (*n* = 306)	10 (3.27)	148 (48.36)	98 (32.03)	50 (16.34)	5.61 ± 3.62

### 3.3 Trajectory of early postoperative nutritional status in patients with gastrointestinal cancer

**Figure 2:**
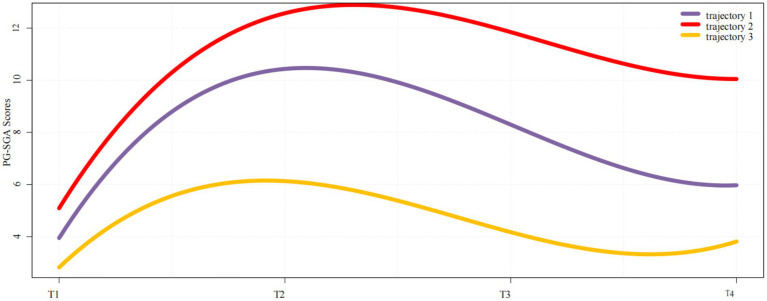
Trajectory of early postoperative nutritional status in patients with gastrointestinal cancer.

Using T1-T4 PG-SGA scores as the observed variables, the number of classes was sequentially increased from 1 to 5 to fit the LGCM model of early postoperative nutritional status in patients with gastrointestinal cancer. The results showed that as the number of classes increased, the values of AIC, BIC, cAIC, ssBIC, and hQIC gradually decreased. When three classes were retained, the BIC value was the lowest, and the average group posterior probabilities were all greater than 0.7. When the number of classes increased to 4 or 5, the BIC values gradually increased, indicating that the model fit did not improve, and the average posterior probabilities of each group decreased. Considering all these parameters, a three-class trajectory model was ultimately determined. Detailed fitting parameters are shown in Table 3. All three trajectory curves showed a trend of first increasing and then decreasing, indicating varying degrees of malnutrition in patients with gastrointestinal cancer in the early postoperative period, as shown in Figure 2. Trajectory 1 included 142 patients (40.46%), who had moderate malnutrition before surgery. Their nutritional status worsened postoperatively but showed some resilience and gradually improved within 3 months, escaping severe malnutrition; therefore, this group was named the “Moderate Malnutrition–Gradual Recovery” group. Trajectory 2 included 41 patients (11.68%), who had moderate malnutrition before surgery, experienced drastic deterioration of nutritional status postoperatively, and remained at a severe malnutrition level 3 months after surgery; thus, this group was named the “Persistent Severe Malnutrition” group. Trajectory 3 included 168 patients (47.86%), who had good nutritional status before surgery, experienced expected moderate malnutrition postoperatively, but rapidly recovered to mild malnutrition within 1 month; therefore, this group was named the “Transient Moderate Malnutrition–Rapid Recovery” group. In addition, the shapes of the retained individual trajectories were determined by fitting cubic, quadratic, and linear terms, progressively eliminating non-significant higher-order polynomials until no further improvement in model fit was observed. The results are detailed in Table 4.

**Table 3:** Fit results of the LGCM for PG-SGA in patients with gastrointestinal cancer.

Type	Average posterior probability					AIC	BIC	cAIC	ssBIC	hQIC
	G1	G2	G3	G4	G5					
1	1					7430.46	7456.696	7461.696	7440.813	7440.266
2	0.947	0.945				6884.171	6941.888	6952.888	6906.946	6905.744
3	0.854	0.892	0.939			6801.82	6891.021	6908.021	6837.018	6835.161
4	0.791	0.873	0.804	0.865		6775.702	6896.385	6919.385	6823.323	6820.81
5	0.800	0.843	0.807	0.862	1.000	6746.703	6898.868	6927.868	6806.746	6803.578

**Table 4:** Trajectory equations of early postoperative nutritional status in patients with gastrointestinal cancer.

Parameter	Trajectory 1				Trajectory 2				Trajectory 3			
	*β*	Se	*t*	*p*	*β*	Se	*t*	*p*	*β*	Se	*t*	*p*
Intercept	−19.609	1.134	−17.30	<0.001	−17.726	2.037	−8.702	<0.001	−12.634	0.719	−17.57	<0.001
t	34.901	1.734	20.13	<0.001	32.859	3.115	10.547	<0.001	23.813	1.100	21.65	<0.001
*t* ^2^	−12.752	0.766	−16.65	<0.001	−11.232	1.376	−8.160	<0.001	−9.506	0.486	−19.56	<0.001
*t* ^3^	1.406	0.102	13.83	<0.001	1.188	0.183	6.500	<0.001	1.145	0.065	17.74	<0.001

### 3.4 Predictive factors for early postoperative nutritional status trajectories in patients with gastrointestinal cancer

#### 3.4.1 Univariate analysis

Using the postoperative early nutritional status trajectory category as the dependent variable, and demographic factors, disease-related factors, handgrip strength, serum albumin, level of sense of coherence, and level of social support as independent variables, a univariate analysis was conducted. The results indicated that gender, age, employment status, education level, monthly household income, cancer type, preoperative neoadjuvant therapy, surgical approach, chemotherapy history, handgrip strength, Level of sense of coherence, and level of social support were statistically associated with the trajectory category. The detailed results are shown in Table 5.

**Table 5:** Univariate analysis of early postoperative nutritional status trajectory categories in patients with gastrointestinal cancer.

Variable	Group	Overall (*n* = 351)	1 (*n* = 142)	2 (*n* = 41)	3 (*n* = 168)	*p*
Gender, *n* (%)						0.01
	Male	253 (72)	104 (73)	37 (90)	112 (67)	
	Female	98 (28)	38 (27)	4 (10)	56 (33)	
Age, *n* (%)						<0.001
	18–39	12 (3)	6 (4)	1 (2)	5 (3)	
	40–59	118 (34)	31 (22)	6 (15)	81 (48)	
	60–79	221 (63)	105 (74)	34 (83)	82 (49)	
Place of residence, *n* (%)						0.21
	City	155 (44)	67 (47)	13 (32)	75 (45)	
	Countryside	196 (56)	75 (53)	28 (68)	93 (55)	
Employment status, *n* (%)						<0.001
	Employed	105 (30)	28 (20)	5 (12)	72 (43)	
	Retired	228 (65)	106 (75)	35 (85)	87 (52)	
	Unemployed	18 (5)	8 (6)	1 (2)	9 (5)	
Education level, *n* (%)						<0.001
	Primary school and below	137 (39)	62 (44)	24 (59)	51 (30)	
	Junior/High school or technical secondary school	177 (50)	69 (49)	11 (27)	97 (58)	
	College and above	37 (11)	11 (8)	6 (15)	20 (12)	
Monthly household income, *n* (%)						0.002
	<3,000	80 (23)	38 (27)	16 (39)	26 (15)	
	3,000–5,000	119 (34)	56 (39)	11 (27)	52 (31)	
	5,000–10,000	84 (24)	29 (20)	8 (20)	47 (28)	
	>10,000	68 (19)	19 (13)	6 (15)	43 (26)	
Comorbidities, *n* (%)						0.622
	No	155 (44)	67 (47)	18 (44)	70 (42)	
	Yes	196 (56)	75 (53)	23 (56)	98 (58)	
Cancer type, *n* (%)						<0.001
	Esophageal cancer	114 (32)	82 (58)	27 (66)	5 (3)	
	Gastric cancer	49 (14)	11 (8)	1 (2)	37 (22)	
	Colorectal cancer	188 (54)	49 (35)	13 (32)	126 (75)	
Cancer stage, *n* (%)						0.256
	I	74 (21)	34 (24)	7 (17)	33 (19)	
	II	129 (37)	52 (37)	16 (39)	61 (36)	
	III	109 (31)	47 (33)	10 (24)	52 (31)	
	IV	39 (11)	9 (6)	8 (20)	22 (13)	
Preoperative neoadjuvant therapy, *n* (%)						<0.001
	No	310 (88)	121 (85)	29 (71)	160 (95)	
	Yes	41 (12)	21 (15)	12 (29)	8 (5)	
Surgical approach, *n* (%)						0.002
	Endoscopic surgery	213 (61)	101 (71)	25 (61)	87 (52)	
	Open surgery	138 (39)	41 (29)	16 (39)	81 (48)	
Radiotherapy history, *n* (%)						0.345
	No	326 (93)	129 (91)	40 (98)	157 (93)	
	Yes	25 (7)	13 (9)	1 (2)	11 (7)	
Chemotherapy history, *n* (%)						0.019
	No	181 (52)	86 (61)	20 (49)	75 (45)	
	Yes	170 (48)	56 (39)	21 (51)	93 (55)	
Sense of coherence		69.47 ± 9.49	71.15 ± 10.04	64.49 ± 9.84	69.27 ± 8.47	<0.001
Level of social support		39.82 ± 6.15	38.24 ± 6.19	37.37 ± 4.91	41.76 ± 5.80	<0.001
Serum albumin		44.41 ± 4.20	44.41 ± 4.87	44.24 ± 4.61	44.45 ± 3.46	0.961
Handgrip strength		29.93 ± 8.19	28.09 ± 7.80	27.40 ± 8.09	32.11 ± 8.01	<0.001

#### 3.4.2 Multinomial logistic regression analysis

Using the postoperative early nutritional status trajectory categories as the dependent variable (with “Trajectory 3” as the reference group), the variables that were statistically significant in the univariate analysis (gender, age, employment status, education level, monthly household income, cancer type, preoperative neoadjuvant therapy, surgical approach, chemotherapy history, handgrip strength, level of sense of coherence, and level of social support) were included as independent variables in a multivariate unordered logistic regression analysis. The results showed that gender, cancer type, handgrip strength, level of sense of coherence, and level of social support were predictive of changes in postoperative nutritional trajectories among patients with gastrointestinal cancer (see Table 6). The likelihood ratio test for the multinomial logistic regression model was *X*^2^ = 268.700, *p* < 0.001, indicating that the model was statistically significant; the maximum pseudo R^2^ was 0.625, showing a good fit; the overall accuracy of predictions made by the multivariate logistic regression model was 77.2%. The recall rates for the three trajectory categories were 73.2, 34.1, and 91.1%, while the specificity rates were 81.8, 98.4, and 79.8%, respectively.

**Table 6:** Multinomial logistic regression analysis of predictive factors for postoperative nutritional status trajectory categories in patients with gastrointestinal cancer.

Trajectory (with ‘3’ as a reference)		*B*	*SE*	*Wald X^2^*	*p*	*OR*	95% confidence interval	
							Upper limit	Lower limit
1	Handgrip strength	−0.175	0.03	34.247	<0.01	0.84	0.792	0.89
	Level of sense of coherence	0.007	0.02	0.108	0.742	1.007	0.968	1.046
	Level of social support	−0.069	0.031	4.849	0.028	0.933	0.878	0.992
	Gender (reference: female)							
	Male	0.926	0.39	5.626	0.018	2.524	1.174	5.424
	Age (reference: 60–79 years)							
	18–39 years old	−0.69	1.537	0.202	0.653	0.502	0.025	10.198
	40–59 years old	−2.201	1.299	2.872	0.09	0.111	0.009	1.411
	Employment status (reference: unemployed)							
	Employed	0.873	0.904	0.931	0.334	2.393	0.407	14.085
	Retired	−0.728	1.524	0.228	0.633	0.483	0.024	9.578
	Education level (reference: college and above)							
	Primary school and below	−0.632	0.635	0.993	0.319	0.531	0.153	1.843
	Junior/High school or technical secondary school	−0.095	0.596	0.025	0.874	0.91	0.283	2.926
	Monthly household income (reference: >10,000)							
	<3,000	−0.93	0.605	2.358	0.125	0.395	0.12	1.293
	3,000–5,000	−0.147	0.493	0.089	0.766	0.863	0.329	2.269
	5,000–10,000	−0.087	0.492	0.031	0.859	0.917	0.35	2.402
	Cancer type (reference: Colorectal cancer)							
	Esophageal cancer	4.988	0.703	50.374	<0.01	146.576	36.974	581.061
	Gastric cancer	0.395	0.541	0.534	0.465	1.484	0.514	4.281
	Preoperative neoadjuvant therapy (reference: Yes)							
	No	−0.126	0.653	0.037	0.847	0.882	0.245	3.169
	Surgical approach (reference: Open surgery)							
	Endoscopic surgery	0.633	0.358	3.125	0.077	1.883	0.934	3.799
	Chemotherapy history (reference: Yes)							
	No	−0.353	0.341	1.075	0.3	0.702	0.36	1.37
2	Handgrip strength	−0.159	0.04	15.834	<0.01	0.853	0.789	0.922
	Level of sense of coherence	−0.066	0.027	5.846	0.016	0.936	0.887	0.988
	Level of social support	−0.09	0.046	3.874	0.049	0.914	0.835	1
	Gender (reference: Female)							
	Male	2.492	0.696	12.827	<0.01	12.091	3.091	47.3
	Age (reference: 60–79 years)							
	18–39 years	−0.91	2.67	0.116	0.733	0.403	0.002	75.491
	40–59 years	−2	2.232	0.802	0.37	0.135	0.002	10.757
	Employment status (reference: unemployed)							
	Employed	1.171	1.474	0.631	0.427	3.227	0.179	58.043
	Retired	0.137	2.58	0.003	0.958	1.146	0.007	180.047
	Education level (reference: college and above)							
	Primary school and below	−0.928	0.873	1.131	0.288	0.395	0.071	2.187
	Junior/High school or technical secondary school	−1.333	0.836	2.547	0.111	0.264	0.051	1.355
	Monthly household income (reference: >10,000)							
	<3,000	−1.131	0.875	1.672	0.196	0.323	0.058	1.793
	3,000–5,000	−0.702	0.795	0.78	0.377	0.496	0.104	2.353
	5,000–10,000	−0.297	0.758	0.153	0.696	0.743	0.168	3.286
	Cancer type (reference: Colorectal cancer)							
	Esophageal cancer	5.33	0.844	39.918	<0.01	206.433	39.509	1078.602
	Gastric cancer	−0.776	1.273	0.372	0.542	0.46	0.038	5.582
	Preoperative neoadjuvant therapy (reference: Yes)							
	No	−0.761	0.738	1.065	0.302	0.467	0.11	1.983
	Surgical approach (reference: Open surgery)							
	Endoscopic surgery	0.294	0.505	0.34	0.56	1.342	0.499	3.608
	Chemotherapy history (reference: Yes)							
	No	−0.573	0.499	1.319	0.251	0.564	0.212	1.499

#### 3.4.3 Decision tree analysis

**Figure 3:**
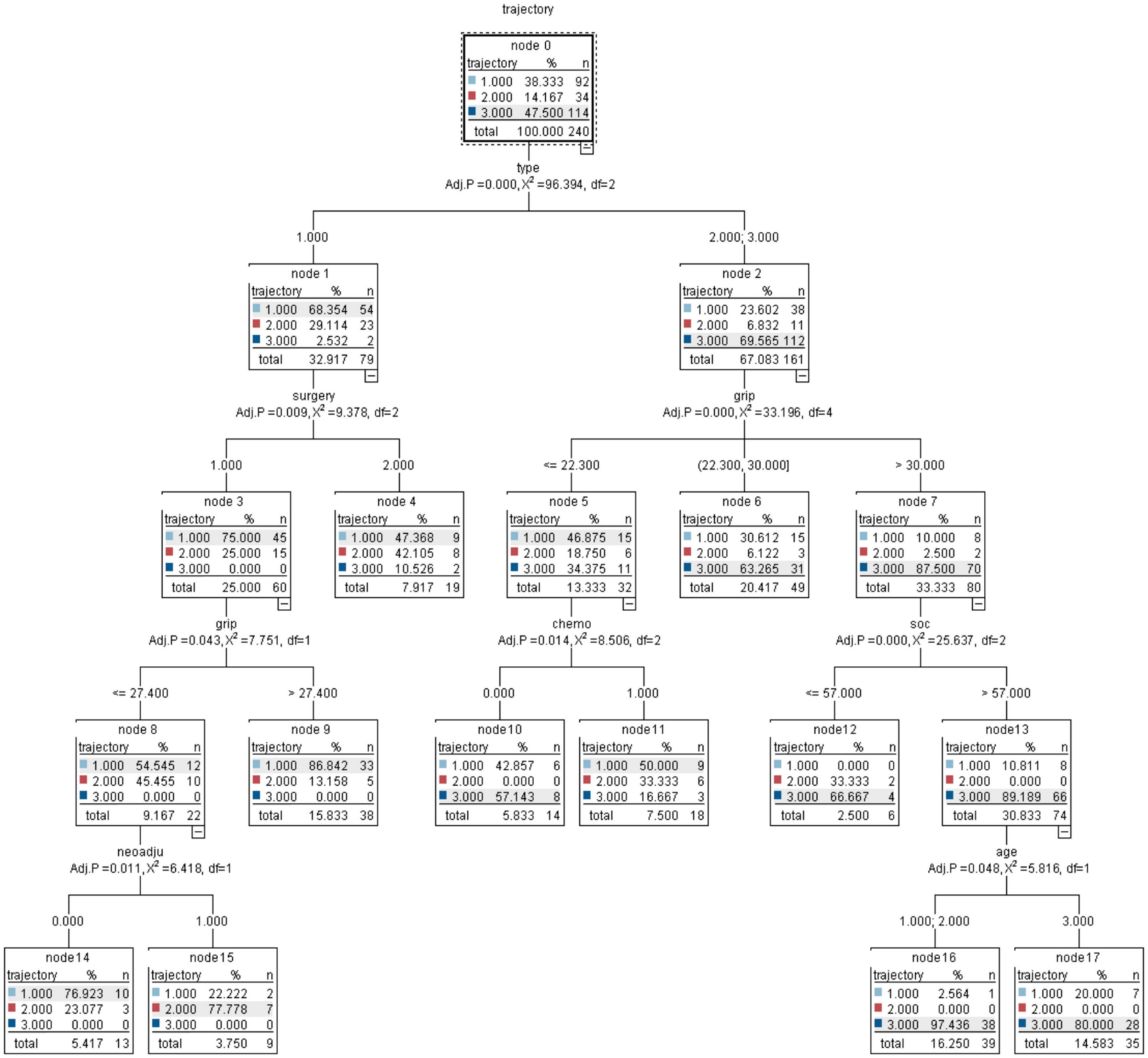
Decision tree model for predicting early postoperative nutritional status trajectory categories in patients with gastrointestinal cancer.

The final results showed that the decision tree consisted of 4 levels with 16 nodes, extracting a total of 7 variables: age, cancer type, surgical approach, chemotherapy history, preoperative neoadjuvant therapy, handgrip strength, and level of sense of coherence, as shown in Figure 3. For the specific meanings of the ‘abbreviations’ shown in Figure 3, see Table 7. The importance ranking of predictive variables was as follows: cancer type > handgrip strength > level of sense of coherence > chemotherapy history > preoperative neoadjuvant therapy > age > surgical approach. The overall accuracy of the decision tree model on the test set was 77.21%, and the recall rates for the three trajectory categories were 60.0, 14.3, and 90.7%, with specificity rates of 86.9, 99, and 61.4%.

**Table 7:** The details of the abbreviations in decision tree model figure.

Abbreviation	Variable	Classification	Content
“type”	Cancer type	1	Esophageal cancer
		2	Gastric cancer
		3	Colorectal cancer
“surgery”	Surgical approach	1	Endoscopic surgery
		2	Open surgery
“chemo”	Chemotherapy history	0	No
		1	Yes
“neoadju”	Preoperative neoadjuvant therapy	0	No
		1	Yes
“age”	Age	1	18–39 years old
		2	40–59 years old
		3	60–79 years old

## 4 Discussion

The nutritional status of patients with gastrointestinal cancer showed significant fluctuations within 3 months after surgery, generally following a trend of initial decline followed by recovery. The nutritional status reaches its lowest point at the T2, with the incidence of moderate to severe malnutrition as high as 97.72%, followed by T3. This finding was consistent with the study by Gonçalves et al. (7), who observed that the incidence of malnutrition in gastrointestinal cancer patients was highest 1 week postoperatively, with an increase in the number of patients with severe malnutrition. Similarly, Zhou et al. (8) used the PG-SGA to assess the nutritional status of 75 patients with esophageal cancer from 1 month to 6 months postoperatively and found that the incidence of malnutrition decreased from 73.4 to 33.5% during this period, with the highest incidence and malnutrition scores occurring at 1 month post-surgery. The first week after surgery is an acute stress period, during which protein catabolism and energy expenditure increase. Concurrently, surgical reconstruction of the gastrointestinal tract alters dietary patterns, and postoperative factors such as pain, limited mobility, and sleep disturbances contribute to significant weight loss and deterioration in nutritional status (27). One month postoperatively marks the early recovery phase, during which gastrointestinal function gradually restores and dietary patterns are being re-established. However, the transition to home-based recovery often involves a lack of professional care. Both patients and family caregivers are in an adjustment period regarding nutritional knowledge and practices, which can easily result in insufficient actual food intake and thereby slow improvement in nutritional status.

In addition, this study found that postoperative early nutritional trajectories among patients with gastrointestinal cancers exhibited heterogeneity. They were categorized based on preoperative nutritional status as well as the degree of subsequent decline and rate of recovery, and named as the “Moderate Malnutrition–Gradual Recovery” group, the “Persistent Severe Malnutrition” group, and the “Transient Moderate Malnutrition–Rapid Recovery” group. It was noteworthy that regardless of the patients’ preoperative nutritional status, a decline in nutritional status occurred within the first 3 months after surgery. This suggested that all patients undergoing gastrointestinal cancer surgery should receive standardized, comprehensive nutritional management, incorporating key steps such as nutrition screening, assessment, diagnosis, intervention, and monitoring throughout the perioperative period. Moreover, clinical nutritional management should be individualized based on the different trajectory patterns. The “Moderate Malnutrition–Gradual Recovery” group accounted for a relatively high proportion (40.46%). Although these patients had some recovery potential, their recovery process was slow and their baseline was weak. Therefore, for this group, the core of clinical nutritional management should focus on “enhanced support and accelerated rehabilitation,” providing proactive nutritional interventions both preoperatively and in the early postoperative period, such as preoperative oral nutritional supplementation and early postoperative combined enteral and parenteral nutrition, to shorten the duration of malnutrition and prevent a decline into the “Persistent Severe Malnutrition” group due to inadequate recovery. The “Persistent Severe Malnutrition” group accounted for 11.68% and may have poorer clinical prognosis, representing the most critical subgroup requiring attention. This study found that their nutritional status was driven by multiple adverse factors, including physiological, psychological, and social dimensions. Thus, conventional nutritional support is often insufficient for these patients. A “multidimensional, precision-based” nutritional management plan must be implemented, and they should be prioritized for home follow-up and resource allocation. The “Transient Moderate Malnutrition–Rapid Recovery” group (47.86%) consists of patients with good preoperative nutritional status. Although expected nutritional decline occurred postoperatively, their recovery was rapid. Nevertheless, the finding that “all patients experience postoperative nutritional decline” indicates that this group could not be neglected either. Systematic nutrition education should be provided to help them understand the universality of postoperative nutritional risk and enhance their self-management capacity. The clinical focus for this group should be on regular monitoring to ensure uninterrupted recovery. In summary, the three nutritional trajectories identified in this study provide a reference for transitioning from a “one-size-fits-all” approach to nutrition support toward a “stratified and precision” management model.

Both the multiple logistic regression model and the decision tree model consistently identified three core and stable predictive variables: cancer type, handgrip strength, and level of sense of coherence. Patients with esophageal cancer, low handgrip strength, and low level of sense of coherence are more likely to be assigned to trajectory 1 or 2. Although few studies had reported on the nutritional status trajectory categories and predictive factors in patients with gastrointestinal cancers, some research exploring the influencing factors of nutritional status in these patients has reached similar conclusions. Song et al. (4), Yang et al. (5), and others found that the incidence of malnutrition in patients with esophageal and gastric cancer is often higher than in those with colorectal cancer, possibly because tumor invasion in the esophagus and stomach can directly affect food intake and digestion. Symptoms such as difficulty swallowing and loss of appetite can lead patients into a chronic catabolic state. In contrast, colorectal cancer has a relatively minor impact on direct food intake, which also aligns with this study’s finding that nutritional trajectories in colorectal cancer patients tend to develop more favorably. Handgrip strength is an effective indicator of muscle function and is closely related to malnutrition. A study focusing on elderly colorectal cancer patients found that GLIM-defined malnutrition is significantly associated with lower handgrip strength (28). Zhang Yinghong and colleagues found that in head and neck cancer patients, lower handgrip strength correlated with higher PG-SGA scores and poorer nutritional status (29). This study found that in decision tree branches, gastric and colorectal cancer patients with a handgrip strength of less than 22.3 kg were more likely to fall into trajectories 1 or 2. A study based on eight Asian community cohorts (including Chinese populations), encompassing 34,265 participants, indicated that the thresholds for low handgrip strength were 30.4 kg for men and 18.1 kg for women (30). The AWGS 2019 recommends diagnostic criteria for low handgrip strength of <28 kg for men and <18 kg for women (31). The 22.3 kg threshold derived in this study, although not gender-specific, falls between the commonly used diagnostic standards for low handgrip strength in men and women, thereby partially validating its clinical relevance as a predictive indicator of nutritional trajectories in this study population. Sense of coherence, proposed by Antonovsky (32), refers to a general, lasting, and dynamic confidence that an individual holds when facing internal and external environmental stimuli, reflecting the overall perception and understanding of life (33). As a key regulatory factor in an individual’s stress response system, sense of coherence significantly modulates clinical recovery by influencing patients’ disease perception and the selection of coping strategies (34). Patients with a high sense of coherence are better able to understand their illness, perceive control, and find meaning. This psychological resource aids in adherence to nutritional therapy and proactive symptom management, thereby improving nutritional outcomes. Yu et al. (35) conducted a prehabilitation intervention combined with positive psychological factors for patients undergoing esophageal cancer surgery and found that, in addition to enhancing patients’ sense of coherence, nutritional status also improved. The positive factor intervention reduced stress responses, thereby mitigating the catabolic effect on protein metabolism. However, factors that we considered to have potential predictive value (such as tumour stage and comorbidities) were not incorporated into the models. Univariate analysis revealed no association between tumour stage or the presence of chronic comorbidities and nutritional status trajectory categories. Consequently, these variables were not included in the multivariate logistic regression and decision tree models. This may be due to the relatively short follow-up period in this study. Tumour stage appeared to have little impact on short-term nutritional status, whereas it was typically a strong predictor of long-term outcomes. For patients eligible for surgery, their preoperative chronic conditions had been properly managed and were usually in a stable, controlled state (for example, blood pressure and blood glucose levels were within target ranges). In such circumstances, the impact of comorbid chronic conditions on short-term nutritional trajectories may not be significant.

However, there were also differences in the predictive variables identified by the two models. In multivariate logistic regression, gender and levels of social support were also important predictive variables, whereas the decision tree tends to predict nutritional status trajectory categories by splitting variables such as chemotherapy history, preoperative neoadjuvant therapy, age, surgical approach. This difference mainly arised from the distinct modeling principles of the two methods: logistic regression focuses on evaluating the independent effects of each variable, while decision trees excel at identifying combinations of predictive variables with interactions through progressive rules, thereby precisely targeting high-risk patient subgroups. This fundamental difference not only led to different variable selections but also affected the performance of the two models in specific predictions. The overall fitting accuracy of logistic regression and decision tree models was 77.2 and 72.1%, respectively, indicating that the two models had similar capabilities in classifying patients at a macro level. However, analysis of the recall rates for each category reveals differences in their predictions. For trajectory 3, both models exhibited relatively high recall rates (Logistic Regression: 91.1%; Decision Tree: 90.7%), indicating that both types of models were highly effective at identifying low-risk patients with good preoperative nutritional status and rapid postoperative recovery. They could accurately ‘filter out’ these patients, thereby avoiding unnecessary medical resource investment. For trajectory 1, the recall rate of Logistic Regression (73.2%) was significantly higher than that of the Decision Tree (60.0%), suggesting that Logistic Regression had an advantage in identifying this intermediate-risk group. As for trajectory 2, both models showed relatively low recall rates (Logistic Regression: 34.1%; Decision Tree: 14.3%), implying that the models (especially the Decision Tree) may miss many patients who were genuinely in the high-risk group. Although both models demonstrate high specificity for predicting trajectory 2 (both exceeding 98%), meaning that once a patient was predicted as high-risk, they were highly likely to indeed be high-risk, the low recall level somewhat limited their clinical predictive value. This was primarily related to the uneven distribution across the three trajectories. In summary, the predictions from Logistic Regression appeared to be more advantageous, but the Decision Tree model offered more intuitive interpretability. Its ‘if…then…’ tree-based rules significantly reduced the cognitive threshold for clinical application. Physicians and nurses could perform rapid risk assessments based on clear rule pathways without needing to understand complex statistical concepts.

This study integrated multivariate logistic regression and decision tree models to jointly identify cancer type, handgrip strength, and sense of coherence as core factors influencing nutritional status trajectories. Logistic regression revealed the impact of independent risk factors such as gender and social support, while the decision tree delineated critical pathways composed of factors like treatment history and age, pointing to specific high-risk subgroups. The conclusions of the two methods were complementary, collectively constructing a comprehensive risk prediction framework. This suggested that future clinical nutrition management could consider developing a stratified management strategy, conducting extensive risk screening based on variables identified by the logistic model, and implementing preventive management for key high-risk groups identified through the detailed pathways revealed by the decision tree, thereby achieving precise prevention and control of malnutrition.

## 5 Limitations

① Limitations in sample representativeness: The study employed a single-center, convenience sampling design. While this approach facilitated the maintenance of follow-up and data quality during the exploratory phase, it may also introduce selection bias, limiting the generalizability of the findings to broader or primary care populations. The findings should be interpreted with caution, and future multi-center studies are warranted. Furthermore, the study excluded patients who developed severe complications, as they were already the primary focus of clinical nutritional management. While this design similarly limited the generalisability of the findings, it allowed us to focus more closely on those groups at nutritional risk who were often overlooked in routine care. ② Limited observation duration: The early follow-up period for this study was set at 3 months post-operation, a critical phase during which the nutritional status of patients with gastrointestinal cancer fluctuated significantly; identifying relevant patterns at this stage was of great importance. However, it did not reveal the trajectory of nutritional status over a longer term. The study is currently undergoing long-term follow-up; the results reported in this paper should be regarded as an interim analysis, intended to inform early clinical decision-making and lay the groundwork for future research. ③ Limitations of measurement tools: While the core assessment tool for nutritional status, the PG-SGA scale, has been validated, some of its items rely on patients’ subjective recall and reporting. To minimise bias arising from differences in patient understanding, we replaced patient self-assessments with standardized interviews conducted by investigators, however, this did not eliminate the issue of recall bias. Objective measures such as serum albumin and handgrip strength were also collected as predictor variables; future studies will need to collect a wider range of objective measures.

## 6 Conclusion

This study, through longitudinal tracking and latent class growth modeling, identified three trajectories of early postoperative nutritional status in patients with gastrointestinal cancer: the ‘moderate malnutrition-gradual recovery’ group, the ‘persistent severe malnutrition’ group, and the ‘transient moderate malnutrition-rapid recovery’ group. This finding provided a reference for shifting from a ‘one-size-fits-all’ approach to nutritional support to a ‘stratified and precise’ management model. Moreover, this study integrated multivariate logistic regression with decision tree models to construct a comprehensive risk prediction framework. Cancer type (esophageal cancer), low level of handgrip strength, and low level of sense of coherence were jointly identified as core predictive factors. Additionally, male and low level of social support were independent risk factors, while decision pathways consisting of chemotherapy history, preoperative neoadjuvant therapy, age, and surgical approach could precisely pinpoint specific high-risk subgroups. Based on this, we could initiate intensified interventions for patients with core risk characteristics, integrating nutritional support, physical strengthening, and psychological counseling. Simultaneously, according to the refined pathways revealed by the decision tree model, preventive management could be implemented for high-risk populations to advance the timing of nutritional interventions.

## Data Availability

The raw data supporting the conclusions of this article will be made available by the authors, without undue reservation.
